# Researches on the Echinococcus in Man and Other Animals

**Published:** 1844-01

**Authors:** 


### ART. XII.

*Recherches sur les Echinocoques, chez l'Homme et chez les Animaux.* Par EUGÈNE LIVOIS, D.M.P. &c.—*Paris*, 1843. 4to, pp. 126.

*Researches on the Echinococcus in Man and other Animals.* By EUGÈNE LIVOIS, D.M.P. &c.—*Paris*, 1843.

THE title of this elaborate treatise will probably not be an attractive one for any but professed helminthologists; but the short analysis which we shall give of its contents will show that they possess a high degree of interest for every medical reader: the author's object being to unveil the real nature of those very obscure and perplexing bodies known under the name of *hydatids* or *acephalocysts*. We cannot but think that in this he has completely succeeded,—that is, if his statements are to be implicitly received; and as he informs us that his researches have been prosecuted under the guidance of M. Rayer, and have been communicated to the Anatomical Society of Paris (of which Dr. Livois is secretary) previously to their publication, we feel much inclined to give credit to them, and the more readily because they tend to reconcile the discrepant accounts given by previous observers.

In giving an account of Dr. Livois' discoveries we shall not follow the order of his memoir; since a large part of this is made up of the statements of the various helminthologists under whose observation the echinococcus has fallen; but we shall endeavour, as briefly as may be, to show in what his discovery consists, by comparing our previous knowledge on the subject with that which we have gained through his researches.

Almost every anatomist is familiar with the bodies formerly termed *hydatids*, but usually designated, since the time of Laennec, as *acephalocysts*; and many discussions have been raised on the question, whether they ought to be considered as independent organisms, or as morbid products of the animal in which they are found. The latter was the opinion of Rudolphi; the former that of most other helminthologists. Our author (with others who have written on the question) considers that two very distinct things have been confounded together,—the real acephalocyst and certain forms of serous cysts; and that these are to be distinguished by their relations to the surrounding tissues,—the true acephalocyst having no adhesion whatever, although inclosed in a more or less distinct cyst which is formed around it, as round any other foreign body; whilst the serous cysts always have some degree of adhesion to the enveloping structures, although this may be but a narrow peduncle. Under this last category he ranges the cysts so frequently found in the plexus choroides, as well as the so-called hydatid mole; which he agrees with Professor Simpson in regarding as a degenerated form of the placenta.

In order to put our readers in possession of the general views previously entertained of the nature of the acephalocyst, we shall quote the account of it given by Mr. Owen, in his article *Entozoa*, in the 'Cyclopedia of Anatomy and Physiology:' "The acephalocyst is an organized being, consisting of a globular bag, which is composed of condensed albuminous matter, of a laminated texture, and contains a limpid colourless fluid, with a little albuminous and a greater proportion of gelatinous substance. The properties by which we recognize the acephalocyst as an independent or individual organized being, are, first, growth by intrinsic power of imbibition; and, secondly, reproduction of its species by germination. The young acephalocysts are developed between the layers of the parent cyst, and thrown off either internally or externally, according to the species." Now on this we would observe, that what are here stated as the characters establishing the *individuality* of the being, are also possessed by every single cell entering into the composition of the organism; for the cells of the secreting and other structures, like those of which the entire embryonic mass is at first composed, grow by their own intrinsic power of imbibition, and have the power of reproducing themselves by germs developed within their cavity. Indeed, Mr. Owen himself has since modified the opinion just quoted; for we find in his recently-published Hunterian Lectures the following passage: "The knowledge that we now possess of the primitive embryonic forms of all animals and of all animal tissues, places us in the position to take a true view of the nature of the acephalocyst. It seems to me to be most truly described as a 'gigantic organic cell,' not as a species of animal, even of the simplest kind." But the evidence in regard to the latter part of the statement is by no means satisfactory. Of the *acephalocystis endogena*, or pill-box hydatid of Hunter, (the species found in man,) Mr. Owen says: "This species is so called from the circumstance of the gemmules being detached from the internal surface of the cyst, where they grow, and in like manner propagate their kind; so that the successive generations produce the appearance described by Hunter and other pathologists. The membrane of the cyst is thin, delicate, transparent, or with a certain pearly semi-opacity; it tears readily and equally in every direction; and can, in large specimens, be separated into laminæ. The vesicles or gemmules, developed in the parietes of the cyst, may be observed of different sizes,—some of microscopic dimensions, others of a line in diameter, before they are cast off." Now, if the statements of Dr. Livois are to be relied on, it would seem that no good microscopic examination has ever been made of these so-called gemmules or granulations; for he represents them, as we shall immediately see, to be of a nature entirely different from the germs of new cysts.

To the previous quotation we shall add Mr. Owen's account of the echinococcus, to the details of which we shall presently have to advert. "The genus *echinococcus* is admitted by Rudolphi into the order *cystica*, less on account of the external globular cyst, which, like the acephalocyst, is unprovided with a head or mouth,—than from the structure of the minute bodies which it contains, and which are described as possessing the armed and suctorious head characteristic of the *cœnuri* and *cystecerci.* It must be observed that Rudolphi does not ascribe this complicated structure to the vermiculi of the human *echinococcus* on his own authority, and speaks doubtfully respecting the coronet of hooklets and suctorious mouths of the vermiculi contained in the cyst of the echinococcus of the sheep, hog, &c. Müller has recently described a species of *echinococcus* voided with the urine by a young man labouring under symptoms of renal disease. The tunic of the containing cyst was a thick white membrane, not naturally divided into laminæ; the animalcules floating in the contained fluid presented a circle of hooklets and four obtuse processes round the head; the posterior end of the body obtuse; some of them were inclosed in small vesicles floating in the large one; others presented a filamentary process at their obtuse end, probably a connecting pedicle which had been broken through." A very similar description has been given by Mr. Quekett and Mr. Curling, of an *echinococcus* found by the latter in cysts developed in the human liver. Mr. Owen gives the following account of his own observations upon the species known as the *echinococcus veterinorum*, of which he examined several individuals soon after they were extracted from the recently-killed animal, a sow, in which they existed in great abundance in cysts in the abdomen. "The containing cysts were composed of two layers, artificially separable; both of a gelatinous texture, nearly colourless, and sub-transparent, the external one being the firmest. The contained fluid was colourless and limpid, with a few granular bodies floating in it; and immense numbers of extremely minute particles applied but not adherent to the internal surface of the cyst. On examining these particles with a high magnifying power, they were seen to be living animalcules of an ovate form, moving freely by means of superficial vibratile cilia, having an orifice at the smaller end, from which a granular and glairy substance was occasionally discharged; and a trilobate depression at the greater and anterior extremity, produced by a retraction of part of the body. I watched attentively and for a long period a number of these animalcules, in the hope of seeing the head completely protruded; but without success. On compressing the animalculæ between plates of glass, a group of long, slender, straight, sharp-pointed spines became visible within the body at its anterior part, and directed towards the anterior depression, precisely resembling the parts described and figured by Ehrenberg as the teeth of the polygastric infusories; the rest of the body was occupied by large clear globules (the stomachs?) and smaller granules. Animalcules thus organized cannot, it is evident, be classed with cystic entozoa, but must be referred to the polygastric infusoria." In Mr. Owen's Hunterian Lectures we find the following additional statement: "The *echinococci* from a small musk-deer, lately dissected at the college, closely resemble those of the hog; but being dead the cellated structure is not indicated, and could not be detected. Each tooth or spine presents an elongated triangular form, a small process extending from the middle of its outer margin, probably for the attachment of the protractor fibres." Dr. Livois, as we shall presently see, has been more fortunate in his opportunities of seeing the animal in its fully-extended form.

The discovery enunciated in the memoir before us is simply this,—that the *echinococcus*, instead of being one of the rarest parasites existing in the human body is among the most frequent, since this entozoon is found in great numbers *in every acephalocyst.* In other words, the true hydatid is nothing else than the containing cyst of a multitude of echinococci. The author considers himself warranted in this generalization, by the fact that after examining upwards of *eight hundred* acephalocysts, he has *in no instance* found the echinococci absent. They are met with, however, in different situations, according to their degrees of development. The granulations or gemmules adherent to the interior of the cyst, are clusters of echinococci, whose head is not yet protruded; but when they have attained their full development they usually separate, and are found floating in the fluid of the sac. In general, the same cyst contains echinococci in both conditions; but sometimes the granulations are wanting, and all the parasites are free, escaping from the sac with the jet of its contained fluid, which is forced out, by the elasticity of its walls, when it is punctured. Hence their existence may easily be overlooked, if the fluid be not collected and examined. The following is Dr. Livois' account of hydatids as they exist in man:

"In the human subject, hydatids usually present themselves under the form of spherical vesicles; but amongst them some are occasionally found of a pear shape, or of an irregular form. It is very rarely that they are found existing solitarily in their cysts, nearly always a more or less considerable number is included in a common cyst. Thus there were 71 in the brain of a girl whose history is reported by Rendtorf; 143 in the kidney of a woman which has fallen under my own observation; and certainly more than 1000 in a vast cyst of the kidney, which M. Rayer was good enough to show me. In this last case the hydatids, which were pressed one against another, offered every variety of size, from that of a grain of millet to that of a large egg; *all these vesicles were free, and did not include any smaller ones.* All those which I examined, and their number was by no means small, contained echinococci; these were very numerous in the larger cysts, but were united in each of the smaller ones into a single group, containing four, five, or six individuals at most. The granulation resulting from this assemblage was very transparent, and was not readily distinguished, on account of its minute size, when looked for through the walls of the hydatid; so that it might easily, even under the microscope, have escaped notice, unless the examination were very carefully conducted. The small worms which were altogether non-adherent, had the same structure and dimensions in every case." (p. 82.)

Hence Dr. Livois concludes that the hydatid, or containing sac, increases in dimensions, as the contained echinococci multiply by reproduction. In regard to the origin of the sac, he does not offer any definite opinion; but speaks of it as *intermediate* between a veritable animal and a morbid product. We have a great dislike to these half-definitions, which seem to help us out of difficulties, whilst they really only substitute one form of words for another. The hydatid must either be an integral part of the organism in which it is contained, resulting from an abnormal development of one of its own cell-germs; or it must be of the nature of a false membrane, a product of the peculiar irritation occasioned by the parasite; or it must be a distinct animal, originating in a germ introduced from without. Now of these three opinions we incline to the second. We cannot suppose the hydatid to be a cell, because it seems to us impossible that the echinococci should find their way into it after it is once formed. We can scarcely imagine it to be a distinct animal, so entirely destitute is it of all claims to be regarded as an independent individual. And, as the observations of Dr. Livois seem to do away with the idea of its reproductive power, we see no valid objection to the idea that it is a product of morbid action. The anatomist can now point to numerous instances in which a pellucid membrane is formed by an action of the subjacent tissue, and without any structural connexion with it,—such is the basement or primary membrane forming the surface of the true skin and mucous membrane; and we do not see any difficulty, therefore, in regarding the hydatid cyst in a similar light.

It is not denied by Dr. Livois that hydatid cysts *may* be found without contained echinococci; this having been reported by Müller in the case already adverted to, and also by Gervais and Gluge. But, in these cases, they were in company with others in which these entozoa presented themselves; and it is quite possible that they may have actually contained entozoa, although these escaped observation from some of the causes just adverted to. But if the existence of true hydatids without echinococci should ever be clearly shown, there would be strong ground for the belief that, in consequence of some unknown influence, the contained entozoa had ceased to exist; since Dr. Livois assures us that, in upwards of 800 examinations, he never found the echinococci absent in a single hydatid.

The hydatids of the lower animals are solitary, instead of being multiplied as in man; that is, in each containing cyst there is but a single hydatid instead of a number. But whilst in man they are but rarely found in several organs at once, or even in several parts of the same organ, the contrary is the case with the lower animals,—hydatids often existing at the same time in the liver and lungs, or in the liver and spleen; and the liver being not unfrequently *riddled* with distinct cysts.

When removed from the body, hydatids soon begin to undergo changes which very much alter their appearance. They lose their transparency, and present an opaline aspect; and the membrane of which they are composed separates into laminæ, of which the exterior is easily torn off; whilst the interior spontaneously falls off in shreds, which float in the contained fluid, giving it a flocculent appearance. Sometimes the whole interior pellicle detaches itself at once; so that we find, as it were, one hydatid contained within another. The various post-mortem alterations which thus result have been attentively studied by M. Cruveilhier. The internal membrane, in detaching itself, carries with it the white granulations, which consist of the adhering echinococci; so that when the cyst alone is examined, no traces of them would be found,—thus affording an additional reason why their existence has so long escaped the notice of observers.

We have, lastly, to consider the structure of the echinococci themselves; and we shall first describe the fully-developed form which they occasionally present, when floating freely in the liquid of the cyst. This may be described as a flattened oval, with a constriction across the middle, and a projection at one extremity. The constriction divides it into what may be termed the head and the caudal vesicle; and the projection borne by the former may be designated as the proboscis. On this proboscis there is a double circular row of hooks, which Dr. Livois states to be forty-four in number; very much resembling that upon the anterior extremity of the *cysticercus*. Behind these hooks, and situated on the head itself, are four suckers, also closely resembling those of the cysticercus. But the caudal vesicle instead of being of great size, as in that of the entozoon, is here no larger than the head itself; it is seen to contain a considerable number of small globular or oval bodies, which are regarded by Dr. Livois as eggs or germs. But the form under which the echinococcus most commonly presents itself, and under which alone it has been recognized by many observers, is very different. The proboscis is drawn back within the head, in a mode resembling the *inversion* of the finger of a glove; and the head itself is drawn within the caudal vesicle, so that the body is reduced to about one half its length, and the circle of hooks on the proboscis can be seen, at about its middle, through the thin parietes of the cavity in which they are imbedded. The anterior extremity now has a funnel-shaped orifice, leading to a canal, which can be traced down as far as the circle of hooks; this canal is due to the inversion of the head and proboscis, as becomes evident from the comparative instance just alluded to. It is in this unfolded state that the echinococci adhere together, in masses usually composed of from fifteen to twenty individuals, so as to form the little granulations that present themselves on the interior of the hydatid,—resembling, according to the description of Dr. Livois, the minute globules of air which adhere to the side of a glass of water. These granulations are easily detached, by agitating the containing cyst; and consequently they cannot be said to have a true adhesion to it. In a short time after the death of the animal in which these parasites occur, *they* seem to share its fate; probably in consequence of an incipient alteration in a state of the fluids. Soon after their death the echinococci begin to lose their transparency; and the circle of hooks, when drawn back within the body, ceases to be distinctly seen. The use of the hooks (in which Dr. Livois describes and figures the lateral projection recently noticed by Mr. Owen,) is evidently to attach the animals to the parietes of the cavity in which they live; that of the suckers is supposed by Dr. Livois to be more temporary, as they serve, in his opinion, to give support to the body whilst the proboscis is being protruded, and to fix it in such a manner that the hooks may be forcibly imbedded in the walls of the sac; which could not be done so long as the animal is floating freely in its fluid. We would suggest whether they may not be sufficiently protruded, in the animals which are grouped in clusters, to serve as the means of their adhesion to one another; this adhesion seems to be pretty strong, since the forms of the individual animals are often greatly changed by the pressure to which they are subjected.

It seems pretty clear that the reproduction of the echinococci takes place by means of ova; but the history of their development has not been traced by Dr. Livois. He states, moreover, that he has never seen any distinct movement in these animals; and on this point he is evidently at issue with Mr. Owen. We would suggest it as by no means impossible that the ciliary movement seen by the latter may be confined to a certain period of development, as it is in the case of many embryos of invertebrated animals.

It only remains to add, that Dr. Livois can recognize no distinction of species among the echinococci of man and of the lower animals; the same forms being presented by all. We commend this very interesting subject to the attentive examination of our pathological microscopists; in the hope that, by their united observations, the real value of this memoir may be speedily determined.

